# Methods to Analyze Time-to-Event Data: The Cox Regression Analysis

**DOI:** 10.1155/2021/1302811

**Published:** 2021-11-30

**Authors:** Samar Abd ElHafeez, Graziella D'Arrigo, Daniela Leonardis, Maria Fusaro, Giovanni Tripepi, Stefanos Roumeliotis

**Affiliations:** ^1^Epidemiology Department, High Institute of Public Health-Alexandria University, Alexandria, Egypt; ^2^Institute of Clinical Physiology (IFC-CNR), Clinical Epidemiology, And Physiopathology of Renal Diseases and Hypertension of Reggio Calabria, Italy; ^3^National Research Council (CNR)–Institute of Clinical Physiology (IFC), Pisa, Italy and Department of Medicine, University of Padua, Italy; ^4^Division of Nephrology and Hypertension, 1st Department of Internal Medicine, AHEPA Hospital, School of Medicine, Aristotle University of Thessaloniki, Thessaloniki, Greece

## Abstract

The Cox model is a regression technique for performing survival analyses in epidemiological and clinical research. This model estimates the hazard ratio (HR) of a given endpoint associated with a specific risk factor, which can be either a continuous variable like age and C-reactive protein level or a categorical variable like gender and diabetes mellitus. When the risk factor is a continuous variable, the Cox model provides the HR of the study endpoint associated with a predefined unit of increase in the independent variable (e.g., for every 1-year increase in age, 2 mg/L increase in C-reactive protein). A fundamental assumption underlying the application of the Cox model is proportional hazards; in other words, the effects of different variables on survival are constant over time and additive over a particular scale. The Cox regression model, when applied to etiological studies, also allows an adjustment for potential confounders; in an exposure-outcome pathway, a confounder is a variable which is associated with the exposure, is not an effect of the exposure, does not lie in the causal pathway between the exposure and the outcome, and represents a risk factor for the outcome.

## 1. Introduction

Survival analysis refers to a family of statistical techniques aimed at analyzing “time-to-event” data and/or assessing the relationship between a given exposure and the occurrence of an outcome after a follow-up period among a cohort of individuals [1]. The Kaplan–Meier (KM) method explores the survival of a population under investigation and/or tests differences in the crude cumulative survival between exposure groups, with a graphical representation of the endpoint occurrence as a function of time [2]. Nonetheless, the KM method has important limitations. First, it does not provide an effect estimate (i.e., a relative risk) or the related confidence interval to compare the survival in different patient groups [3]. Second, it does not permit the adjustment of confounders in etiological research or predictors in prognostic research [4]. Finally, the KM method requires data categorization, so calculation of the incremental increase (or decrease) in the relative risk of a given event associated with one unit (or any number of units) increase in the candidate risk factor is not possible [5]. These limitations can be approached by Cox regression analysis, in which the dependent variable is the incidence rate of a specific event and the independent variables are risk factors or predictors that the investigators use to explain or predict the study endpoint.

In 1972, Sir David Cox wrote an article describing an extension of KM analysis to incorporate patients' baseline characteristics, such as age, clinical history, or exposure to certain factors, and time [6]. The Cox regression model is also known as proportional hazards regression analysis. It is a semiparametric method because there is no assumption about the distribution of survival times, but it assumes that the effects of different variables on survival are constant over time (proportionality assumption) and additive over a particular scale [7].

A preliminary step when testing the relationship between a candidate risk factor and the incidence rate of an endpoint using the Cox regression model is to assess the proportionality assumption of the risk linked to the same risk factor; such an assumption considers that the hazard ratio (HR) associated with the risk factor must be constant over time. For example, if the risk factor is a binary variable (e.g., the presence of diabetes) and the survival curves of the two groups are crossed, it implies that the HR (diabetic vs. nondiabetic patients) is not constant over time [8].

The HR and its 95% confidence interval are calculated from the Cox regression model. The HR is the ratio of the two hazard rates of a given event in treated versus untreated patients or exposed versus unexposed individuals, as well as the magnitude of the hazard rate change when a given continuous variable (e.g., age or systolic blood pressure) increases by, for example, one or two units over a continuous scale [9]. The purpose of this article is to explain the basic concepts of Cox regression analysis and its application to clinical research by providing a series of examples derived from the literature.

### 1.1. The Cox Equation

The Cox regression model is based on the hazard function. Mathematically, the Cox model is written as follows [10]:

(1) $$\begin{array}{c} H\left(t\right)=H_{0}\left(t\right)\times \exp\left[b_{1}x_{1}+b_{2}x_{2}+\cdots ..b_{k}x_{k}\right]. \end{array}$$

where *x*_1_ ⋯ *x*_*k*_ represents the predictor variables and *H*_0_(*t*) is the baseline hazard at time *t*, which is the hazard of an individual having the predictors set to zero. By computing the exponential of the regression coefficient “*b*_1_ ⋯ *b*_*k*_” (directly provided by the software), we can calculate the HR of a given risk factor or predictor in the model. For example, if the risk factor *x*_1_ is dichotomous and it is codified “1” if present (exposed) and “0” if absent (unexposed), the expression exp^(*bi*)^ (where exp = 2.7183) can be interpreted as the estimated increase in the HR of the event in patients with the risk factor compared to those without the same risk factor; this is applied by assuming exposed and unexposed patients are similar for all the other covariates included in the model. If the risk factor is a continuous variable and it is directly related to the incidence rate of a given event (e.g., age in years as a risk factor for mortality), the HR will be interpreted as an increase in the hazard rate of death due to a 1-year increase in age. Thus, if a study reports a HR of 1.03 for age, it implies that the hazard rate of the event of interest increases by 3% for each year increase in age. If the risk factor is a continuous variable and it is inversely related to the incidence rate of a given event (e.g., albumin in g/dL, a protective biomarker of nutritional status, as a predictor of death), the HR associated with albumin is interpreted as the decrease of the hazard rate of the event due to a 1 g/dL increase in albumin. Thus, if a study reports a HR of albumin of 0.85, it implies that the hazard rate of the event decreases by 15% for each 1 g/dL increase in albumin.

## 2. The Hazard Ratio and Incidence Rate Ratio

**Figure 1:**
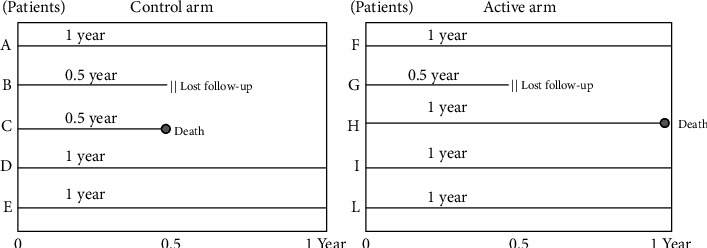
Hypothetical example of an incidence rate calculation (see text for more details).

We interpret the HR similarly to the incidence rate ratio (IRR); thus, the best way to describe this measure is to provide an example of an IRR calculation [11]. Consider a hypothetical randomized clinical trial to test the efficacy of a generic drug X on 1-year mortality among cardiovascular disease patients. For simplicity, we will explain this concept based on data from five patients in the intervention arm and five patients in the control arm. During the follow-up period (1 year), in the control arm, patient B was lost to follow-up, patient C died after 6 months, and patients A, D, and E completed the follow-up period (Figure 1(a)). In the intervention arm, patient G was lost to follow-up, patient H died at the end of the study (i.e., after 1 year), and patients F, I, and L completed the follow-up period (Figure 1(b)). To assess the risk of mortality in each study arm, we calculated the probability of death in both the control (1/5 = 0.20 or 20%) and the active (1/5 = 0.20 or 20%) arms; for this, the risk ratio will equal to 1. It is evident that the risk ratio calculation does not capture the fact that the event of interest (death) occurred later in the active (at 1 year) than in the control arm (at 6 months). Therefore, we should calculate the incidence rate to estimate the frequency of death in the two study groups. In this case, the incidence rate of death in the two study arms is the ratio between the number of deaths and the total person-time (i.e., the sum of the times of observation of all patients) in each arm. The incidence rate among the control group is 1/4 = 0.25 deaths/person-year (i.e., 25 deaths per 100 persons-year), and the incidence rate among the intervention group is 1/4.5 = 0.22 deaths per person-year (i.e., 22 deaths per 100 persons-year). The incidence rate calculation shows that mortality occurs earlier in the control group compared to in the active group; this is illustrated by the higher incidence rate among control patients than active patients (25 versus 22 deaths per 100 persons-year). The IRR is calculated by dividing the incidence rate of death among the intervention group and the incidence rate of death among the control group (22/25 = 0.88); the IRR of 0.88 means that the probability of death occurring first is 12% lower among patients in the intervention arm than in those in the control arm. Given the similarity between the two measures, the HR can be interpreted in exactly the same way as the IRR.

## 3. Example 1

A hypothetical cohort study was performed on 200 chronic kidney disease patients aged ≥65 years. We aimed to calculate the HR of myocardial infarction among patients with high oxidative-low-density lipoprotein (LDL) versus low oxidative-LDL and test whether this relationship is independent of a history of diabetes mellitus (see Table 1).

The hazard rate of myocardial infarction as a function of oxidative-LDL, using the Cox equation, can be written as follows:

(2) $$\begin{array}{c} H\left(t\right)=H_{0}t*\exp\left[{}^{b*smoking\left(0=no;1=yes\right)}\right]. \end{array}$$

*H*_0_ *t* refers to the risk component due to time, and exp^[*b*∗*oxidative* − *LDL*]^ is the risk component due to oxidative-LDL (codified as “0” for low oxidative-LDL and “1” for high oxidative-LDL). *H*_0_*t* and the “*b*” coefficient are estimated by the maximum likelihood function [12]. In this example, we are interested in knowing the HR of myocardial infarction in patients with high versus low oxidative-LDL, so we focus on the “*b*” coefficient and ignore *H*_0_*t*. In our example, the value of “*b*” (as calculated using statistical software) is 1.69.

By applying the general formula, we can calculate the HR using the following equation:

(3) $$\begin{array}{c} \text{HR}=\exp^{b},\\ \text{HR}=2.71831^{.69},\\ \text{HR}=5.44. \end{array}$$

This means that the HR of myocardial infarction is 5.44 times higher in patients with high oxidative-LDL versus those with low oxidative-LDL. Then, we test the potential confounding effect of diabetes mellitus on this relationship. Diabetes is a confounder because it is associated with both death (patients with high oxidative-LDL have a higher risk of mortality compared to those with low oxidative-LDL) and oxidative-LDL (i.e., the prevalence of diabetes is significantly higher in patients with high oxidative-LDL than those with low oxidative-LDL). The Cox equation now includes two variables: oxidative-LDL and diabetes:

(4) $$\begin{array}{c} H\left(t\right)=H_{0}t*\exp\left[{}^{b1*\text{oxidative}‐\text{LDL}+\text{b}2*\text{diabetes}}\right]. \end{array}$$

The introduction of diabetes into the Cox model including oxidative-LDL yields a reduction in the regression coefficient of oxidative-LDL from 1.69 to 0.92. In the same equation, the regression coefficient of diabetes is 0.18.

Thus, the HR of oxidative-LDL adjusted for diabetes is calculated as follows:

(5) $$\begin{array}{c} \text{H}{\text{R}}_{\text{oxidative}-\text{LDL}}=2.7183^{\left(0.92\right)}=2.51. \end{array}$$

Therefore, the diabetes-adjusted HR of myocardial infarction is 2.5 times higher in patients with high oxidative-LDL than in those with low oxidative-LDL.

By using the same formula, we can calculate the oxidative-LDL-adjusted HR of diabetes.

(6) $$\begin{array}{c} \text{H}{\text{R}}_{\text{diabetes mellitus}}=2.7183^{\left(0.18\right)}=1.20. \end{array}$$

Thus, the oxidative-LDL-adjusted HR of myocardial infarction is 20% higher in diabetics than in nondiabetics.

## 4. Example 2

A prospective population-based study by Spoto et al. [13] was conducted to assess the effect of gamma glutamyl transferase (GGT) on the risk of all-cause mortality among Italian people older than 65 years and free from liver disease (*n* = 1,038). The median follow-up duration was 9 years (range 0.15–10.5 years). During the follow-up period, 401 subjects died. Crude and adjusted Cox regression analyses were performed to test the possible association between serum GGT and all-cause mortality. Multiple Cox regression models included serum GGT as a continuous variable as well as traditional risk factors (age, sex, smoking status, diabetes, LDL cholesterol, systolic blood pressure (SBP), and past cardiovascular (CV) events), hepatic disease-related factors (transaminases (AST/ALT), alkaline phosphatase (Alk_P), and alcohol consumption), body mass index (BMI), hemoglobin, oxidized LDL, C-reactive protein (CRP), homocysteine, and creatinine clearance. (Table 2) The authors constructed a model with adequate statistical power by introducing approximately one covariate into the model for every 22 patients who died. As shown in Table 2, based on the crude analysis, a 20 U/L increase in serum GGT signaled a parallel 10% increase in the risk of all-cause mortality (HR 1.10, 95% CI 1.03–1.18, and *P* = 0.007). An adjustment for all potential confounders did not materially change the strength of the link between GGT and mortality (HR 1.11, 95% CI 1.02–1.21, and *P* = 0.02, Table 2). The study concluded that serum GGT is an independent risk factor for all-cause mortality among the elderly population.

## 5. Example 3

**Figure 2:**
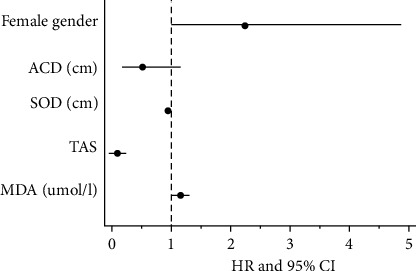
Forest plot of the hazard ratios for the risk factors associated with visual field progression in the paper by Li et al. [14].

A prospective longitudinal cohort study was conducted among 94 patients with primary angle-closure glaucoma (PACG) and 89 normal controls, who were followed up for 2 years (periodic visits every 6 months); the study is aimed at identifying the baseline oxidative stress-related factors predicting the progression of PACG [14]. In univariate Cox regression analyses, the female gender (*P* = 0.048), anterior chamber depth (*P* = 0.049), superoxide dismutase (SOD, *P* = 0.005), total antioxidant status (TAS, *P* < 0.001), and malondialdehyde (MDA, *P* = 0.008) were identified as significant predictors of the study end point. In a multivariable regression model, the females had a two-fold risk for developing PACG compared to males (HR = 2.228, 95% CI: 1.013–4.897, and *P* = 0.046), independently of all other factors. Higher levels of MDA (HR (1 *μ*mol/l): 1.010; 95% CI: 1.001–1.018, *P* = 0.015), lower levels of SOD (HR (1 U/mL): 0.983; 95% CI: 0.971–0.994, and *P* = 0.003), and TAS (HR: 0.041; 95% CI: 0.008–0.218, and *P* < 0.001) were independently associated with the progression of PACG, as measured with the visual field (Figure 2) [14].

## 6. Example 4

In a study of patients with end-stage renal disease, Zoccali et al. [15] investigated the relationship between systolic dysfunction (as assessed by left ventricular ejection fraction, LVEF) and the incidence rate of fatal and nonfatal cardiovascular (CV) events. In a multiple Cox regression model, a 1% decrease in LVEF signaled a 4% increase in the hazard rate of fatal and nonfatal CV events (HR: 1.04, 95% CI: 1.02–1.07, and *P* = 0.001) independently of a series of potential confounders, including age, gender, antihypertensive treatment, smoking, CRP, and SBP. To assess whether the link between LVEF and fatal and nonfatal CV events could be explained by the confounding effect of the left ventricular mass index (LVMI, a variable closely related to both LVEF and fatal and nonfatal CV events), the authors included this biomarker in the multiple Cox regression model. The relationship between LVEF and fatal and nonfatal CV events remained statistically significant after adjusting for LVMI. The authors concluded that studying myocardial contractility by echocardiography provides etiological information independently of LVMI and other risk factors in ESRD.

## 7. Conclusions

The Cox regression analysis is a fundamental statistical method for addressing etiological and prognostic hypotheses. It is based on estimating the HR associated with a specific risk factor or predictor for a given endpoint. Interpretation of the HR crucially depends on the units of measurement of each variable in the model. The number of covariates tested by the Cox method must account for the number of patients with the event of interest. The standard Cox regression method allows for an investigation of the effect of one or more variables (covariates) on the “time-to-first-event” analysis. An assessment of proportional hazards is a prerequisite to fitting a Cox regression model. In survival analysis, both Kaplan–Meier analysis and Cox regression methods are used to address etiological and prognostic hypotheses in clinical and epidemiological research.

## Figures and Tables

**Table 1:** Baseline characteristics of 200 chronic kidney disease patients.

Patients	Age (years)	Oxidative-LDL (0: low and 1: high)	Time (year)	Myocardial infarction (0: no and 1: yes)	Diabetes mellitus (0: no and 1: yes)
1	65	0	15	0	0
2	80	0	9	0	1
3	67	1	1	1	1
4	90	1	16	1	1
5	77	1	20	0	0
….	…..	…..	…..	…..	…..
200	83	0	3	0	1

**Table 2:** Multiple Cox regression models of all-cause mortality.

		Crude model		Adjusted model
	Units of increase	Hazard ratio (95% CI), *P* value		Hazard ratio (95% CI), *P* value
GGT	20 U/L	1.10 (1.03–1.18), *P* = 0.007		1.11 (1.02–1.21), *P* = 0.02
Age	1 year			1.13 (1.11–1.15), *P* < 0.001
Gender	Male gender			1.26 (0.98–1.63), *P* = 0.08
Current smokers	Yes/no			1.93 (1.43–2.63), *P* < 0.001
BMI	1 kg/m^2^			1.01 (0.98–1.04), *P* = 0.49
LDL cholesterol	1 mg/dl			0.99 (0.98–0.99), *P* = 0.007
C-reactive protein	1 *μ*g/mL			1.01 (1.01–1.02), *P* = 0.006
SBP	1 mmHg			1.01 (0.99–1.01), *P* = 0.17
Alk_P	1 U/L			1.00 (1.00–1.01), *P* = 0.01
Hemoglobin	1 g/dL			1.04 (0.96–1.13), *P* = 0.32
Alcohol consumption	1 g/day			0.99 (0.98–0.99), *P* = 0.03
AST	1 U/L			1.01 (0.99–1.04), *P* = 0.26
ALT	1 U/L			0.98 (0.96–0.99), *P* = 0.01
Diabetes mellitus	Yes/no			1.17 (0.85–1.61), *P* = 0.35
Creatinine clearance	1 ml/min/1.73 m^2^			1.00 (0.99–1.01), *P* = 0.80
Oxidized LDL	1 U/L			1.01 (0.99–1.02), *P* = 0.18
Past CV events	Yes/no			1.48 (1.15–1.92), *P* = 0.002
Homocysteine	1 *μ*mol/L			1.02 (1.00–1.03), *P* = 0.002

## Data Availability

All data supporting the results of this paper are presented.
